# Proteomic Analysis of Follicular Fluid in Polycystic Ovary Syndrome: Insights into Protein Composition and Metabolic Pathway Alterations

**DOI:** 10.3390/ijms252111749

**Published:** 2024-11-01

**Authors:** Janusz Przewocki, Adam Łukaszuk, Grzegorz Jakiel, Izabela Wocławek-Potocka, Karolina Kłosińska, Jolanta Olszewska, Krzysztof Łukaszuk

**Affiliations:** 1 Institute of Mathematics, University of Gdansk, 80-308 Gdańsk, Poland; 2 iYoni App—For Fertility Treatment, LifeBite, 10-763 Olsztyn, Poland; luka@gumed.edu.pl; 3 Edinburgh Medical School, College of Medicine and Veterinary Medicine, The University of Edinburgh, 47 Little France Crescent, Edinburgh EH16 4TJ, UK; a.z.lukaszuk@sms.ed.ac.uk; 4 Invicta Research and Development Center, 81-740 Sopot, Poland; grzegorz.jakiel1@o2.pl; 5 First Department of Obstetrics and Gynaecology, Centre of Postgraduate Medical Education, 01-004 Warsaw, Poland; 6 Department of Gamete and Embryo Biology, Institute of Animal Reproduction and Food Research, Polish Academy of Sciences, 10-748 Olsztyn, Poland; i.woclawek-potocka@pan.olsztyn.pl; 7 Department of Obstetrics and Gynecology, Oncological Gynecology and Gynecological Endocrinology, Medical University of Gdansk, 80-214 Gdańsk, Poland; kklosinska@uck.gda.pl; 8 Department of Obstetrics and Gynecology Nursing, Medical University of Gdańsk, 80-210 Gdańsk, Poland; jolanta.olszewska@gumed.edu.pl

**Keywords:** follicular fluid, proteomics, mass spectrometry, PCOS, apolipoprotein A-I, myosin light polypeptide 6, alpha-2-macroglobulin, alpha-1-antitripsin

## Abstract

This study explores the proteomic composition of follicular fluid (FF) from women undergoing oocyte retrieval for in vitro fertilisation (IVF), with a focus on the effects of polycystic ovary syndrome (PCOS). FF samples were collected from 74 patients, including 34 with PCOS and 40 oocyte donors. Proteomic profiling using machine learning identified significant differences in protein abundance between the PCOS and control groups. Of the 484 quantified proteins, 20 showed significantly altered levels in the PCOS group. Functional annotation and pathway enrichment analysis pointed to the involvement of protease inhibitors and immune-related proteins in the pathophysiology of PCOS, suggesting that inflammation and immune dysregulation may play a key role. Additionally, HDL assembly was identified as a significant pathway, with apolipoprotein-AI (APOA1) and alpha-2-macroglobulin (A2M) as the major proteins involved. Notably, myosin light polypeptide 6 was the most downregulated protein, showing the highest absolute fold change, and may serve as a novel independent biomarker for PCOS.

## 1. Introduction

The ovarian follicle is a fundamental component of the human reproductive system that provides a highly specialised microenvironment for oocyte growth, maturation and possible ovulation. Within each follicle, an oocyte develops in close association to a collection of follicular fluid (FF), known as the antrum. Both of these structures are surrounded by multiple layers of somatic cells, which regulate the process of oogenesis by secreting hormonal signalling molecules and other bioactive compounds. Due to such structural organisation, the FF acts as an important mediator of molecular exchanges between the developing oocyte and its surrounding somatic cells. Indeed, human FF is known to contain a diverse range of proteins, growth factors, metabolites and immune cells, all of which have been previously found to influence oocyte viability as well as its fertilisation potential [1]. The biochemical composition of FFs, however, has a highly dynamic nature, being constantly influenced by a combination of local ovarian stimuli and systemic factors, such as hormones, inflammation, and metabolic conditions [2]. Hence, since FF is highly responsive to physiological fluctuations and exerts a direct effect on oogenesis, the analysis of FF composition might serve as a window into a patient’s ovarian function and reproductive potential. The insights derived from FFs may be especially valuable in exploring the causes of female infertility, which is estimated to have increased globally by 14% between 1990 and 2017 [3]. This phenomenon may be partially attributed to delayed attempts at pregnancy and the associated age-related changes [4]. Many high-income countries have seen a rise in the proportion of women ambivalent about childbearing, often postponing the decision to start a family until their thirties [5]. However, many women in this age group are also affected by the rising rates of obesity and insulin resistance, which are involved in the pathogenesis of polycystic ovary syndrome (PCOS). Unfortunately, PCOS can have a deleterious effect on oocyte quality, directly impacting the reproductive potential of a female patient and causing infertility.

PCOS is a multifaced endocrine disorder characterised by hyperandrogenism, insulin resistance, and chronic anovulation, all of which contribute to a disrupted ovarian microenvironment [6]. The influence of these pathological factors can also be seen in the case of FF that exhibits significant changes to its composition in patients with PCOS [2]. Notably, the reduced effectiveness of insulin action has been previously demonstrated to be associated with a downregulated expression of insulin-like growth factor binding proteins (IGFBPs) in the FF, with IGFBP-1 being particularly affected [7]. These molecules target insulin-like growth factors (IGFs), which play a key role in ovarian function by promoting folliculogenesis, stimulating granulosa cell proliferation, and facilitating oocyte maturation. Hence, the level of FF IGFPBs has a direct influence on the availability of IGF-1, meaning that the decreased expression of IGFPBs seen in PCOS patients with hyperinsulinemia results in elevated free IGF-1. Due to their ability to bind and activate insulin receptors, molecules of IGF-1 enhance the steroidogenic activity within ovarian follicles, causing a further increase in the synthesis of androgens and exacerbating the hyperandrogenic state [8]. Such an abnormal hormonal environment disrupts the delicate balance of oestrogen and progesterone production, which is crucial for proper follicular development and endometrial receptivity. The clinical impact of these changes is especially evident in PCOS patients undergoing IVF, in whom lower implantation rates and higher miscarriage rates are observed compared to non-PCOS women [9].

The negative effect of PCOS on the FF composition are further exacerbated by the altered lipid metabolism associated with this condition. As reported in previous studies, the accumulation of free fatty acids (FFAs) in the FF has the potential to interfere with mitochondrial function in granulosa cells, which comprises energy metabolism and hampers their ability to support oocyte maturation [10]. Therefore, the ovarian follicles of PCOS patients affected by FFA-mediated lipotoxicity are likely to produce oocytes of lower quality, as mitochondrial activity is essential for proper spindle formation and chromosomal segregation in oocytes [11]. Furthermore, high levels of FFAs in FF have also been associated with an increased inflammatory state within the follicular microenvironment. Indeed, a study by Lai et al. demonstrated FFAs to trigger the production of reactive oxygen species (ROS) and induce inflammasome activation in FF samples obtained from women with PCOS [12]. The resultant oxidative stress results in a substandard microenvironment for folliculogenesis and oocyte maturation, hence influencing the overall reproductive success of women diagnosed with PCOS. Indeed, according to published data, patients with PCOS undergoing IVF procedure have a lower fertilisation rate compared to women without the condition [13].

Thus, given the pronounced negative impact of PCOS on female fertility and the success rates of assisted reproductive technology (ART), it seems that the ability to identify reliable biomarkers that reflect the various components of this complex condition’s pathomechanism could provide substantial clinical benefits. Such a tool could help clinicians to more accurately predict IVF outcomes in patients with PCOS, potentially allowing for a more personalised approach to fertility treatment that accounts for the complex nature of the condition.

## 2. Results

### 2.1. Clinical Data of Donors

The study population included a total of 74 patients, who underwent oocyte retrieval in preparation for in vitro fertilisation. The indication for the procedure was either PCOS (n = 34) or self-expressed willingness to donate oocytes (n = 40). Statistics summarising the clinical characteristics of all participants are displayed in Table 1.

### 2.2. Proteomic Analysis Using Machine Learning Techniques

In order to investigate the potential influence of PCOS on the proteomic composition of follicular fluids, the measured protein abundances were used to construct Random Forest classifiers that aimed to differentiate samples obtained from each of the two study subgroups. The algorithm was designed to perform classification within datasets based on the Gini Impurity Index, which quantifies impurity for any set of samples *Q* according to the following equation:

$$I\left(Q\right)=\sum_{class\in \{healthy,pcos\}}p_{class}\left(1-p_{class}\right)$$

where $p_{class}$ indicates the relative frequency of samples with a clinical characteristic class (healthy donor or patients with PCOS) present in set *Q*.

The Random Forests were composed of 50 decision trees, each having a maximum depth of their splitting points limited to three to avoid overfitting. To further increase the models’ generalisation ability, the constructed trees were pruned by applying a stopping criterion that ensured a minimum of 30 samples to be present in every leaf node (no further hyperparameter optimisation was performed since our goal is to identify the most important predictors and not to find models with the maximal predictive power). Additionally, the samples were assigned weights that were inversely proportional to the absolute size of their respective classes (control group or PCOS group), which allowed to minimise bias towards overrepresented classes that the model might have otherwise exhibited.

The models were trained iteratively, using Recursive Feature Elimination with Cross-Validation (RFECV, see Section 4.5) to progressively remove non-informative features. During this process, *protein scores* were generated. A group 20-fold cross-validation scheme was applied, ensuring that samples from each patient appeared only once in each test set, across 20 distinct folds. The algorithm was executed for 30 cycles, with the final protein scores representing the sum of scores generated in each cycle. The performance of the predictive models in each cycle was assessed using the balanced accuracy metric.

To evaluate the statistical significance of the data derived from the Random Forest algorithm, confidence intervals (CIs) at a 95% confidence level were calculated using the bootstrapping technique. This involved generating 10,000 resamples through simple sampling with replacement. For each resample, the median protein abundance was computed within each group. The statistic used in the bootstrapping process was the difference in medians between the groups. To make the sampling distribution more symmetric, the data were first transformed using the logarithm. Then, in order to avoid pseudoreplication, samples from each follicular fluids were made independent by calculating medians over biological repetitions for each sample.

The results of the analysis (see Table 2) revealed that, amongst the 484 quantified proteins, approximately 20 displayed markedly higher scores when compared to the remaining ones (median protein score was equal to 6.0 with IQR being 9.5). Since the utilised scoring system reflects the capacity of the variable to discriminate between classes, it can be inferred that these proteins are associated with the greatest changes in FF abundance between the clinical subgroups (PCOS and healthy donors). Furthermore, when the proteomic composition of FF samples obtained from healthy patients was taken as a reference, several disease-specific alterations in protein concentrations were identified.

Interestingly, in the follicular fluids of participants with PCOS ($FF_{PCOS}$), nearly all statistically significant changes in the proteomic composition were positive, indicating a general trend of elevated protein concentrations in FFs affected by this condition. The sole exception was myosin light polypeptide 6, which was the only significantly downregulated protein. While plasma protease C1 inhibitor and antithrombin-III were also found at lower concentrations, their individual changes were not statistically significant; however, they were deemed significant in the context of a multi-variable Random Forest model.

**Figure 1:**
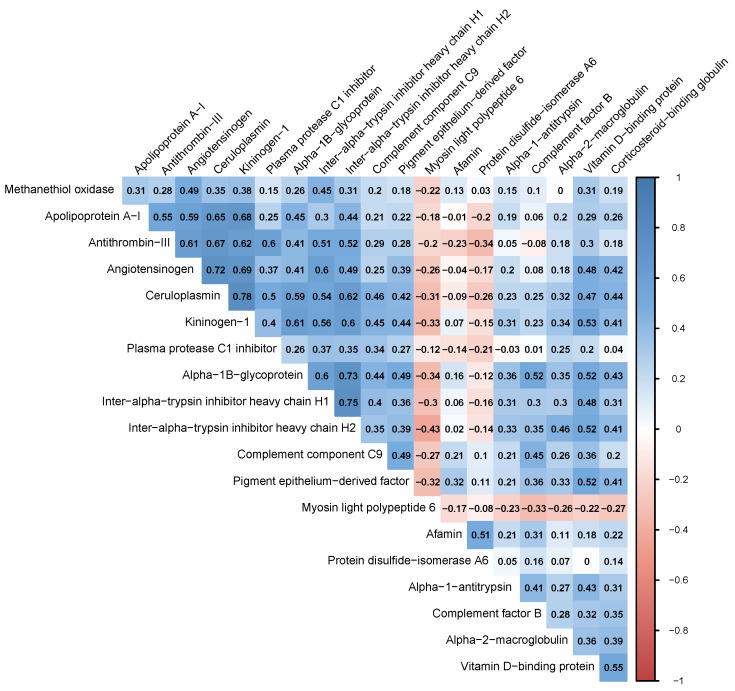
Correlation matrix calculated with the robust algorithm based on winsorisation [14].

Among the 20 upregulated proteins identified in the $FF_{PCOS}$ samples, the most significant increases in abundance were observed in angiotensinogen, complement factor B, and ceruloplasmin. Myosin light polypeptide 6 was the only protein found to be significantly downregulated, and it showed a slight negative correlation with other proteins, suggesting its potential as an independent biomarker for PCOS (see Figure 1).

The final Random Forest classifier, trained on the abundances of 20 selected proteins in FF samples, demonstrated a significant improvement over the baseline (i.e., the random classifier). Its balanced accuracy, confirmed through cross-validation, reached 64%. However, it is important to note that although mass spectrometry can measure multiple protein abundances simultaneously, it is susceptible to considerable measurement error. Therefore, the performance of the model could certainly be improved with more accurate measurements of the selected proteins.

### 2.3. Functional Annotation and Pathway Enrichment Analysis Using PANTHER and StringDB

In this section, we present a comprehensive functional analysis of the identified proteins using various bioinformatics tools. First, protein classification was performed using the PANTHER v19.0 database [15] to categorise the proteins based on their annotated protein class. Next, pathway enrichment analysis was conducted with PANTHER to identify overrepresented pathways in the Reactome database [16], shedding light on the biological processes potentially impacted. Finally, protein–protein interaction (PPI) networks were analysed using StringDB [17] to uncover potential interactions, providing deeper insights into their roles in PCOS.

The results presented in Table 3 show that the majority of the identified proteins (50%) were classified as protein-binding activity modulators, and notably, all of these are annotated in PANTHER as protease inhibitors. This group consists of alpha-1-antitrypsin, corticosteroid-binding globulin, angiotensinogen, plasma protease C1 inhibitor, inter-alpha-trypsin inhibitor heavy chains H2 and H1, alpha-2-macroglobulin, antithrombin-III, pigment epithelium-derived factor, and kininogen-1.

In the context of polycystic ovary syndrome (PCOS), these protease inhibitors provide valuable insights into the syndrome’s pathophysiology. PCOS is characterised by chronic low-grade inflammation [18,19], and the elevated activity of protease inhibitors may reflect a response to the inflammatory state [20]. This increase likely represents a compensatory mechanism to counterbalance excessive proteolytic activity, helping to preserve tissue integrity and prevent damage to ovarian follicles.

This distribution underscores the functional diversity of proteins in the sample, with a notable emphasis on the regulatory and immune-related roles. Additionally, the presence of defence/immunity proteins (15%) further supports the idea that immune system dysregulation is a contributing factor in PCOS.

While cytoskeletal proteins and chaperones make up a smaller proportion of the proteins, they still play essential roles in maintaining cellular structure and proper protein folding—critical processes for normal follicular function that may be disrupted in PCOS.

The results of the reactome pathway enrichment analysis are presented in Table 4. It reveals several biologically relevant pathways that are significantly overrepresented in the context of PCOS, providing important insights into the syndrome’s underlying mechanisms. Among the pathways showing the highest fold enrichment are *HDL assembly* and *plasma lipoprotein assembly*. These pathways involve two key proteins: apolipoprotein A1 (APOA1) and alpha-2-macroglobulin (A2M). A2M plays a critical role by inhibiting the conversion of pro-apolipoprotein A1 into lipid-binding APOA1, a process that involves the removal of six N-terminal residues. In vitro studies have demonstrated that tetrameric A2M concentrations can suppress this reaction [21].

**Figure 2:**
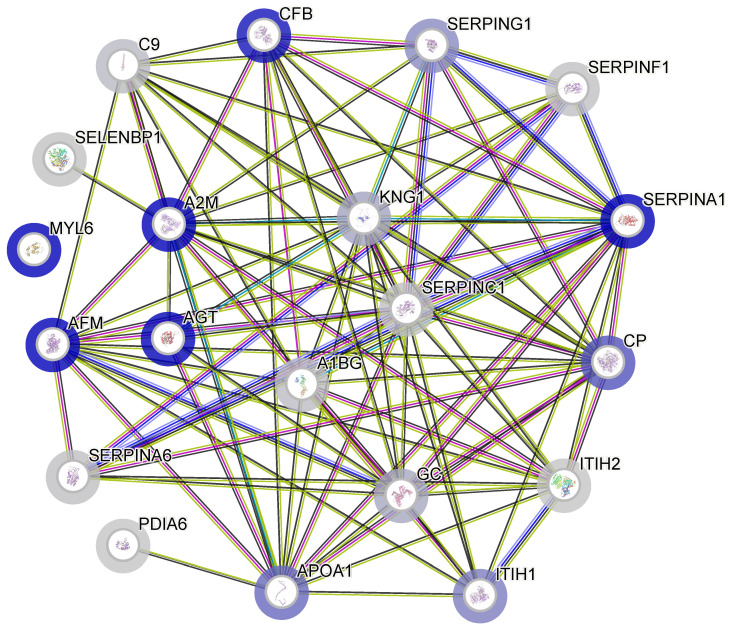
A protein–protein interaction network functional enrichment analysis created using STRING v.12.0. Predicted interactions are summarised using coloured lines: gene co-expression by a black line, gene neighbourhood by a green line, experimental evidence by a purple line, database evidence by a light blue line, and text-mining evidence by a yellow line. The proteins are labelled by the names of their corresponding genes (see Table 2).

Finally, the identified set of 20 FF proteins was screened for the presence of significant protein–protein interactions using the STRING Network Software (version 12.0). Such relationships are often a major predictor of the effect that a protein exerts on the cellular metabolism and could, therefore, provide insight into the potential functional systems and biochemical pathways relevant to the follicular fluid composition. The graph presented in Figure 2 highlights the intricate network of interactions between several proteins that may be relevant in the context of polycystic ovary syndrome (PCOS). Several central nodes in this graph, such as A2M (alpha-2-macroglobulin), SERPINA1 (alpha-1-antitrypsin), and AGT (angiotensinogen), are notable for their roles in inflammation, immune regulation, and protease inhibition. These proteins form central hubs in the network, suggesting they have multiple interactions and are likely key players in PCOS pathophysiology. It is important to note that the only protein that seemed to be independent of the others as indicated by the correlation matrix—myosin light polypeptide 6 (MYL6)—does not exhibit any known interactions with other proteins.

## 3. Discussion

The majority of proteins that exhibit differential abundance in FF samples in our study are associated with HDL particles. Indeed, the number of proteins that have been linked to HDL in various studies approaches 1000 [22]. However, not all findings have been consistently confirmed across multiple studies. As of today, according to the HDL Proteome Watch webpage [23], the association of 285 proteins has been confirmed in at least 3 out of 51 studies. Out of the proteins we were able to quantify in our study, 177 (36.5%) are associated with HDL particles. However, HDL-associated proteins are overrepresented among those showing differential abundance in our study. Of the 20 proteins exhibiting differential abundance according to our statistical model, 17 (85%) are HDL-associated. The three proteins not known to be HDL-associated are methanethiol oxidase, myosin light polypeptide 6, and protein disulfide-isomerase A6.

Follicular fluid consists of secretions from oocytes, granulosa cells within the follicles, and thecal cells surrounding the follicle, as well as plasma transudate filtered through the basal membrane. The basal membrane acts as a filter, allowing the passage of proteins with relatively low molecular weight. The composition of follicular fluid is similar to that of plasma with respect to low-molecular-weight substances. For example, electrolyte concentrations are the same as those in plasma [24], while proteins with a molecular weight above 100 kDa are found at progressively lower concentrations [25].

The authors of the study [26] who examined mouse follicles using their developed ‘in vivo cryotechnique’, demonstrated that the variability in protein concentrations in follicular fluid depends on the molecular weight. For example, albumin, being a low-molecular-weight protein (approximately 69 kDa), freely passes through the blood–follicle barrier. In contrast, medium-weight proteins such as IgG1 (approximately 150 kDa), inter-alpha trypsin inhibitor (approximately 220 kDa), and fibrinogen (340 kDa) show significantly reduced concentrations within ovarian follicles. For high-molecular-weight proteins like IgM (approximately 900 kDa), concentrations are the most restricted.

HDL constitutes a heterogeneous family of particles as reflected by the existence of various classification schemes [27]. However, all HDL particles contain the apolipoprotein A-I, and some also contain apolipoprotein A-II (the fraction containing both proteins has a molar ratio of 2:1 relative to ApoA1:ApoA2 [28]). It is known that HDL particles are variable in size. Gofman identified two main subclasses: HDL2, which is less dense (1.063–1.125 g/mL), and HDL3, which is denser (1.125–1.21 g/mL). The molecular weight of HDL2 is around 360 kDa, while HDL3 is closer to 175 kDa[27]. These figures suggest the reduced permeability of HDL through the blood–follicle barrier. Indeed, research has shown lower ApoA1 concentrations in FF than in plasma [29].

Apolipoprotein A-I can also occur in a lipid-free or lipid-poor form, sometimes referred to as preβ1-HDL [30], which accounts for approximately 5% of its plasma concentration. Despite the fact that some LDL, VLDL, and chylomicron particles may contain ApoA1 [27,31], this proportion is small, and the concentration of ApoA1 strongly correlates with the number of HDL particles [32]. This suggests that ApoA1 concentrations in the blood are strongly tied to the number of HDL particles, and this link is likely even stronger for ApoA1 in follicular fluid since larger lipoproteins are found in much smaller quantities in FF compared to HDL. For example, the main component of LDL, ApoB, has a molecular weight of approximately 500 kDa[33], making it less likely to easily pass through the blood–follicle barrier, although there is some evidence of LDL and VLDL being present in FF at very low concentrations [34].

Studies on PCOS have shown that lipid profiles tend to worsen in these conditions [35,36,37], often with lower HDL-cholesterol levels. However, our findings revealed a statistically significant increase in ApoA1 levels in FF samples from patients with PCOS. Moreover, many other HDL-associated proteins showed alterations compared to the control group in this disease condition. These changes cannot be explained solely by shifts in the lipid profile of the studied patients.

The other apolipoproteins quantified in our FF study were apolipoprotein F, apolipoprotein A-II, apolipoprotein A-IV, apolipoprotein C-II, apolipoprotein C-III, and apolipoprotein D. A separate analysis revealed that only ApoA4 and ApoC3 were statistically significant based on their bootstrap confidence intervals. These apolipoproteins did not appear in the primary analysis because their predictive value was lower compared to the selected proteins.

Apolipoprotein C-III was found to have a strong correlation with ApoA1 (r=0.67). While ApoC3 is predominantly associated with VLDL particles [27] and only present in small amounts in HDL particles, the low VLDL concentration in FF suggests that changes in ApoC3 levels are likely linked to HDL particles. Additionally, ApoC3 levels were, on average, higher in PCOS patients compared to the control group, with an effect size of 0.27 (0.06–0.41), slightly greater than that of ApoA1 (see Table 2). This points to the possibility of altered HDL particle structure in PCOS patients. Recently, ApoC3 contained in HDL was found to be associated with a decrease in insulin sensitivity in a prospective observational study involving a cohort of 864 healthy volunteers [38]. Similar association was also found for PCOS patients [39].

Authors of the study [40] also found ApoA4 to be increased in the follicular fluid. Intrestingly, we were able to confirm more findings of this study. Namely, we also found increased concentrations of kininogen-1, alpha-1B-glycoprotein, and antithrombin.

Pathway enrichment analysis revealed an overrepresentation of proteins involved in the “Regulation of IGF transport and uptake by IGFBPs” pathway, suggesting an important role in PCOS pathogenesis. Several protease inhibitors identified as significant in our study may be directly associated with this pathway. Notably, IGFBP-3, a critical modulator of IGF bioavailability, is cleaved by enzymes such as plasmin, kallikreins, and thrombin. We hypothesise that compensatory mechanisms in PCOS may aim to reduce the activity of these enzymes, particularly those responsible for IGFBP-3 cleavage. Supporting this hypothesis, our data align with known protease inhibitor interactions: plasmin is inhibited by alpha-2-macroglobulin [41], while thrombin is regulated by antithrombin [42]. Additionally, kallikrein, which not only promotes plasmin activity [43] but also acts as an IGFBP protease [44], may play a dual role in this context. Its lower levels, observed in our study, could contribute to increased kininogen abundance, likely due to reduced cleavage [45], which is consistent with our findings. The data suggest a complex regulatory network influencing IGFBPs and protease activity in PCOS.

Another surprising finding of our study is the association of the protein myosin light polypeptide 6 with PCOS. According to our data, this protein shows a negative correlation with all other identified biomarkers except intercellular adhesion molecule 1. This is significant because most of the proteins found in the study show a positive correlation (this is especially evident with apolipoproteins). Additionally, it is worth noting that no interaction with any of the identified biomarkers was shown in the STRING database. Myosin light polypeptide 6 is the product of the MYL6 gene, which is believed to be non-muscle-myosin-2-specific [46]. Its previous association with PCOS cannot be found in the existing literature.

## 4. Materials and Methods

### 4.1. Patient Recruitment

The study was conducted in 2019 at the Medical University of Gdansk (Poland) in partnership with Invicta fertility clinics. For the purposes of the investigation, egg cells were obtained from female patients who planned to undergo in vitro fertilisation procedure due to previous history of PCOS. The control group consisted of oocytes obtained from women who voluntarily expressed their willingness to become donors. Each participant subsequently underwent a set of routine screening tests to confirm their eligibility. During this process, females aged less than 18 or more than 35 years as well as those with a history of abnormal fertilisation results in previous cycles were excluded from the study. Due to the pandemic and the associated challenges in sourcing materials, the sample collection period was extended to early 2023.

In total, 74 patients were selected for egg cell harvesting (see summary in Table 5). The follicular fluids were collected exactly 36–37 h after the artificial induction of final maturation through the activation of LH/CG receptors. They were then immediately transferred to the embryologist, who continuously reported on the cumuli obtained up to that point (clusters of granulosa cells from the released ovarian thalamus that may contain an oocyte). If no oocyte was obtained from a given follicle, the attempt was repeated by rinsing the follicle with the same fluid and retrieving it again. After the procedure, the samples were filtered through a 5 μm mesh at room temperature to remove the erythrocytes, white blood cells, and granulosa cells. The fluid was collected and stored separately from each follicle at −20 °C for further analysis. The oocytes were kept separately and labelled with the same number as the collected and frozen fluid. Fluids were collected separately, and embryos arising from specific follicles were cultured separately. The number of follicles collected ranged from 2 to 11; however, we tested fluids from 2 to 3 randomly selected fluids from each patient. Overall, the analysis involved 213 follicular fluids (112 fluids from PCOS patients and 110 fluids from donors).

The described experiments were conducted as a part of a project titled “Identification of Biomarkers of Early Embryonic Development and Pregnancy”, following an approval granted by the Independent Bioethics Committee of the Medical University of Gdansk (decision 62/2016). Prior to recruitment, each participant was familiarised with the design of the study and then asked to provide written consent, which also included permission to publish the results of the analysis conducted on the donated egg cells, given that patient anonymity would be fully maintained.

### 4.2. Oocyte Retrieval and Culture

#### 4.2.1. Stimulation

To facilitate oocyte retrieval, all participants were treated according to ‘short protocol stimulation’, which was described previously by Łukaszuk et al. (2013) [47]. Prior to the start of stimulation, patients were screened with a series of ultrasound and hormonal tests so as to exclude the presence of dominant follicles and confirm that the peripheral blood hormone levels were within the following limits: oestradiol < 50 pg/mL, LH < 6 mIU/mL, and progesterone < 0.5 ng/mL. Having ruled out the possibility of the premature recruitment of a dominant follicle, gonadotropins were administered to achieve ovarian stimulation. Menopausal gonadotropin preparations (Menopur, Ferring, Parsippany, NJ, USA) were chosen for this purpose, as they produce equal FSH and LH activity. The precise dosing regimen used in the case of each patient was determined based on their baseline AMH levels and ranged between 150 and 225 IUper day. In addition to the gonadotropin therapy, 0.05 mgof triptorelin was administered subcutaneously from the first day of stimulation. After 7 days of treatment, the gonadotropin dose was modified in preparation for oocyte retrieval. The stimulation protocol was halted once the presence of at least 3 follicles with a diameter measuring more than 18 mm was confirmed ultrasonographically. Finally, 36 h before planned oocyte retrieval, each patient received a 5000 IUdose of hCG (Pregnyl, MSD, Bloomington, IN, USA) intramuscularly to induce the last stage of oocyte maturation.

#### 4.2.2. Oocyte Retrieval and Collection of Samples

In order to collect oocytes following stimulation, donors were put under brief general anaesthesia using a combination of propofol and fentanyl. The oocytes were first visualised on a GE Voluson P6 ultrasound with an IC-9-RS vaginal transducer and subsequently collected through a disposable oocyte retrieval needle (Genomed, Löhne, Germany). Samples of follicular fluid were then taken from the harvested ovarian follicles and transferred to the embryologist who, upon microscopy-based examination, provided regular updates on the number of cumuli—defined as clusters of granulosa cells from the ovarian follicle that might contain an oocyte—obtained so far. If an egg cell could not be isolated from a given follicle, the collection of a sample was attempted again by rinsing the follicle with the same fluid and retrieving it again. The fluid samples were further filtered through a 5 μm mesh (at room temperature) to remove any remaining erythrocytes, white blood cells, or granulosa cells. After processing, the follicular fluids were frozen at −20 °C.

### 4.3. Sample Preparation

The collected samples were sought to be investigated using qualitative and quantitative measures, which included the preparation of a spectral library for SWATH-MS quantification studies. As summarised in Table 6, several steps were undertaken to ensure optimum sample preparation and instrument performance throughout the analysis. In brief, after thawing, the follicular fluid was centrifuged at 1000× *g* for 10 min to separate all morphological structures. To allow for the greatest number of proteins to be included in the library, MARS 14 column (Agilent, Santa Clara, CA, USA) was utilised to immunodeplete proteins that were present at the highest concentrations. Digestion of the protein material was achieved by applying FASP (trypsin) on a Microcon with a 30 kDacut-off membrane (Merck-Millipore, Burlington, MA, USA) according to a standardised protocol (1:50 enzyme-to-protein weight ratio). The MED-FASP procedure included three consecutive digestions with the following proteolytic enzymes: LysC (1:50), trypsin (1:100), and chymotrypsin (1:100) (all obtained from Promega Corporation, Madison, WI, USA). Furthermore, to minimise bias associated with slight chromatogram shifts, calibration peptides (iRT peptides) were added to each analysed sample in a 1:10 standard-to-sample ratio. The iRT (index retention time) kit (Biognosys, Zurich, Switzerland) was used in the case of all samples intended for SWATH-MS spectral library preparation or SWATH-MS quantification, hence allowing for the calibration of the retention time parameter during the procedures. Overall, by applying this method, a collection of more than 2000 proteins was generated for further analysis (Table 6).

### 4.4. LC-MS/MS Measurements and Quantitative Data Processing

The LC-MS/MS analysis was performed on the TripleTOF 5600+ hybrid mass spectrometer with DuoSpray Ion Source (AB SCIEX, Framingham, MA, USA) and Eksigent microLC (Ekspert MicroLC 200 Plus System, Eksigent, Redwood City, CA, USA). First, the samples were applied onto the LC columns utilising a CTC Pal Autosampler with an injection volume of 5 μL(CTC Analytics AG, Zwinger, Switzerland). A mixture of 0.1% (*v*/*v*) formic acid in water (Buffer A) and ACN (Buffer B) was then added to ensure ionisation control as well as pH stability during analysis. The LC procedure was performed using a pre-designed system, in which the molecular constituents of each sample were separated within ChromXP C18CL columns (3 μm, 120 Å, 150 × 0.3 mm; Eksigent, Redwood City, CA, USA) set at a gradient of 8–40% Buffer B per 30 minand a flowrate of 5 μL/min. A positive ion mode was applied during all measurements. To minimise associated error, the constructed system was controlled automatically using the Analyst TF 1.7.1 software (AB SCIEX, Framingham, MA, USA).

Data-dependent acquisition (DDA) analyses involved an initial 250 msTOF survey scan in the 400–1000 Dam/z range, which was followed by a 100 msProduct Ion scan in the territory of 100–1500 Dam/z. This resulted in an average cycle time equal to 2.3 s. A selection of the 20 most suitable ion candidates—with a charge state ranging between 2 and 5—was subsequently used for collision-induced dissociation (CID) fragmentation with a rolling collision energy. In cases when a former ion was detected twice, it was excluded for a period of 5 s. All SWATH-MS analyses were conducted using a looped product ion mode. First, 25 variable-width windows were constructed based on equalised ion frequency distribution with a m/z range of 400–1000 Da[48]. The quantification of collision energy was performed only for ions with charges between +2 and +5. At the beginning of each cycle, initial high-sensitivity survey scans were performed in the range of 400–1000 Dautilising an accumulation time of 50 ms. This was followed by high-sensitivity product ion scans with an accumulation time of 40 ms, resulting in a total cycle time of 1.1 s.

To allow for spectral library construction, the SwissProt database (version 26.07.2019; 20,428 entries) was searched according to the Paragon algorithm with the use of a ProteinPilot 4.5 Software (AB SCIEX, Framingham, MA, USA). The target database was limited to the Homo sapiens species and merged with the iRT standard sequence together with the following parameters: TripleTOF 5600 instrument, cysteine alkylation by iodoacetamide, trypsin enzyme digestion, focus on biological modifications, thorough ID search effort, and detected protein threshold [Conf] >10%. The resulting group file was loaded into MS/MS All with SWATH-MS Acquisition MicroApp 2.01 in PeakView 2.2 (AB SCIEX, Framingham, MA, USA) to automatically create a spectral library with parameters set to allow modified peptides and exclude shared peptides. The library was then processed with the SWATH-MS measurements derived from the samples. Calibration of the retention time parameter was performed manually using iRT kit peptides. The maximum number of peptides per protein was set to 6, with extracted ion chromatogram (XIC) parameters at a 10 min extraction window width and a 75 ppmXIC width. Normalisation was performed at two points during the analytical process—initially, the spectra from individual samples were normalised in MarkerView based on the total area sums, and subsequently, SWATH-MS intensities were normalised across all samples in Perseus.

### 4.5. Random Forest Algorithm with Recursive Features Elimination

The Random Forest is a powerful algorithm primarily used to classify samples based on selected features (in our case, protein abundances). This machine learning technique excels in classification tasks by leveraging multiple decision trees to improve predictive accuracy and handle complex datasets, such as those generated in proteomics studies. Based on the premise that statistical significance is related to effect size, we can use the Random Forest, in tandem with a feature-selection technique (in our case, Recursive Feature Elimination), not only as a predictive model but also as a tool to detect statistically significant differences in protein abundances across different groups of samples. The application of the Random Forest in analysing mass spectrometry data is well documented in the literature. This method has been used to identify patterns and classify data based on protein abundances, aiding in the discovery of biomarkers and the understanding of biological processes. The algorithm operates by constructing multiple decision trees during training, each trained on a different random subset of samples and based on a random subset of features. By combining the predictions of these individual trees through averaging, it enhances predictive accuracy and mitigates the risk of overfitting. A single decision tree within the Random Forest ensemble is a structure composed of nodes and edges, resembling a tree. Each node represents a subset of the training samples. The tree begins with a root node representing the entire training set for this particular tree. Each node can have up to two child nodes, which are classified into two types: split nodes and leaf nodes. Split nodes, having two child nodes, divide the subset of samples into two parts corresponding to each child node. Leaf nodes have no child nodes. Here, we present an overview of how a single decision tree is created:

**Initialisation:** The tree starts with a root node containing a random subset of samples.

**Best feature selection:** The algorithm searches for the optimal feature to split the samples. The split creates two subsets: one with higher values and one with lower values. The feature and the splitting threshold are chosen to maximise the homogeneity within the two subsets. Ideally, all samples in a subset belong to the same class. This homogeneity is measured using the Gini impurity, where an impurity of zero indicates perfect homogeneity.

**Splitting:** Each subset from the split becomes a new node in the decision tree. This process is repeated recursively for each node.

**Stopping criterion:** The recursion stops when one of the following conditions is met:

- The maximum tree depth is reached.
- The number of samples at the current node falls below a certain threshold.
- Further splitting does not significantly improve the chosen metric.

**Leaf nodes:** Once a stopping criterion is met, the current node becomes a leaf node and is assigned a probability distribution based on the distribution of labels within that node.

An advantageous characteristic of Random Forests is their ability to rank features by assigning importance to each feature. Typically, the Mean Decrease in Impurity (MDI) is used as an estimate of feature importance. This measure investigates how the Gini impurity changes when splitting the samples within each node (i.e., the difference between the Gini impurity of the parent node and the mean impurity of the child nodes). The MDI of each feature can be calculated and averaged over the whole Random Forest Classifier, ranking the features according to their importance in the prediction process. Feature importance, as described above, can be used to identify statistically significant proteins. We utilise Recursive Feature Elimination with Cross-Validation (RFECV) for this purpose. The strategy involves fitting multiple Random Forests while progressively eliminating the least important features. Here is a brief description of the algorithm:

1. The samples are divided into folds, with some used as the training set and others as the test set. The test set is used to calculate the accuracies of the models trained on the training set.
2. At each stage, features with the lowest importance are eliminated until the optimal number of features $n_{features}$ is found—i.e., the number of features that maximises the accuracy on the test set.
3. All features except the $n_{features}$ most important ones are eliminated.
4. This process is repeated multiple times.
5. During this process, each feature that is not eliminated is assigned a score. The highest scores correspond to features that are “persistent” (i.e., not eliminated by the algorithm for a long time). Features with the highest scores are considered statistically significant.

The details of the above algorithms are covered more extensively in our previous publication [49].

## 5. Conclusions

This study offers significant insights into the proteomic changes in follicular fluid from patients with polycystic ovary syndrome when compared to healthy oocyte donors. Our analysis revealed notable differences in the abundance of key proteins, indicating that the proteomic profile of FF in PCOS is generally characterised by elevated protein levels, most of which are associated with HDL particles. This finding implies a substantial role of the HDL metabolism in the pathophysiology of PCOS, despite HDL particles being somewhat limited in their movement across the blood–follicle barrier due to their size.

Interestingly, the lipid profile of PCOS patients typically shows lower HDL-cholesterol levels, contrasting with the increased ApoA1 levels identified in our study. This discrepancy points to the influence of additional factors contributing to the altered metabolic landscape in PCOS. In addition to the lipid metabolism and transport pathways, our findings highlight the compensatory mechanisms involving the increased activity of certain protease inhibitors, as well as the significance of immune and inflammatory processes in the syndrome’s pathophysiology, particularly regarding the complement system, where the most pronounced changes are observed in complement factor B.

One unexpected finding is the association of myosin light polypeptide 6, a cytoskeletal protein not previously linked to PCOS. Its negative correlation with other proteins—especially those related to HDL—suggests a novel and distinct role in the disease.

In conclusion, the proteomic analysis of FF in PCOS reveals a complex interplay of proteins involved in immune regulation, HDL assembly, and protease inhibition. These findings enhance our understanding of the molecular mechanisms underlying PCOS and present potential biomarkers for future diagnostic and therapeutic applications. A limitation of this study is the inability to determine whether the observed changes in the follicular fluid proteome are driven by systemic or local factors, as many of the proteins detected are serum filtrates. Further research is needed to validate these proteins and pathways in larger cohorts using more accurate methodologies, which could refine the predictive models for PCOS diagnosis and improve disease management.

## Figures and Tables

**Table 1:** A summary of the clinical characteristics of the recruited patients. Values are presented as means (standard deviation).

Variable	PCOS	Control	*p*-Value
No. subjects	30	43	
Age (years)	27.5 (2.4)	27.1 (2.3)	n.s.
BMI (kg/m^2^)	24.3 (3.8)	21.7 (3.3)	p<0.05
AMH (ng/mL)	4.8 (2.3)	2.8 (1.5)	p<0.05
Inhibin B (pg/mL)	53 (34.3)	62.3 (30.8)	n.s.
Day 3 basal FSH (mIU/mL)	6.3 (2.6)	6.3 (4.3)	n.s.
Day 3 basal LH (mIU/mL)	8.7 (3.3)	7.2 (5.3)	n.s.
Day 3 basal oestradiol (pg/mL)	56.2 (28.2)	43.2 (30.4)	n.s.
DHEAS (μg/dL)	211 (93)	226 (72.5)	n.s.
Testosterone (nmol/L)	1.8 (1.6)	1.5 (1.7)	n.s.
SHBG (nmol/L)	66 (36.2)	74.2 (35.4)	n.s.
AFC	24.4 (7.2)	16.7 (6.2)	p<0.05

Abbreviations: AFC—antral follicles count; AMH—anti-Mullerian hormone; BMI—body mass index; DHEAS—dehydroepiandrosterone-sulfate; FSH—follicle-stimulating hormone; LH—luteinizing hormone; SHBG—sex hormone-binding globulin; n.s.—not significant.

**Table 2:** Proteins identified as significant by the Random Forest Classifier with Recursive Feature Elimination (RFECV) algorithm (i.e., having an outlying protein score). Point estimates are presented along with the 95% bootstrap confidence intervals. Statistically significant bootstrap intervalsare indicated by an asterisk (*).

Uniprot ID	Gene Name	Protein Name	Score	Log_2_ FC pcos
P60660	MYL6	Myosin light polypeptide 6	330	−0.61 (−0.90,−0.34) *
P43652	AFM	Afamin	330	0.21 (0.13,0.36) *
P01009	SERPINA1	Alpha-1-antitrypsin	330	0.23 (0.14,0.32) *
P01023	A2M	Alpha-2-macroglobulin	330	0.26 (0.09,0.49) *
P01019	AGT	Angiotensinogen	324	0.41 (0.13,0.67) *
P00751	CFB	Complement factor B	301	0.36 (0.12,0.55) *
P00450	CP	Ceruloplasmin	210	0.35 (0.09,0.58) *
P02647	APOA1	Apolipoprotein A-I	178	0.18 (0.08,0.31) *
P19827	ITIH1	Inter-alpha-trypsin inhibitor heavy chain H1	146	0.19 (0.09,0.34) *
P05155	SERPING1	Plasma protease C1 inhibitor	128	−0.07 (−0.19,0.04)
P01042	KNG1	Kininogen-1	93	0.24 (0.10,0.35) *
P02774	GC	Vitamin D-binding protein	83	0.28 (0.13,0.41) *
P02748	C9	Complement component C9	59	0.18 (0.06,0.32) *
P04217	A1BG	Alpha-1B-glycoprotein	55	0.18 (0.013,0.34) *
P08185	SERPINA6	Corticosteroid-binding globulin	51	0.29 (0.14,0.42) *
P01008	SERPINC1	Antithrombin-III	49	−0.07 (−0.20,0.10)
P36955	SERPINF1	Pigment epithelium-derived factor	48	0.29 (0.10,0.58) *
Q13228	SELENBP1	Methanethiol oxidase	46	0.33 (0.15,0.50) *
P19823	ITIH2	Inter-alpha-trypsin inhibitor heavy chain H2	44	0.23 (0.11,0.33) *
Q15084	PDIA6	Protein disulfide-isomerase A6	44	0.34 (0.085,0.51) *

**Table 3:** Protein classes based on PANTHER annotations.

Protein Class	Number of Proteins	Percentage of Proteins
transfer/carrier protein	3	15%
defence/immunity protein	3	15.0%
cytoskeletal protein	1	5.0%
chaperone	1	5.0%
protein-binding activity modulator	10	50.0%
metabolite interconversion enzyme	1	5.0%
unclassified	1	5.0%

**Table 4:** Pathway enrichment analysis results for pathways catalogued in the reactome database.

Reactome Pathway	Fold Enrichment	FDR
HDL assembly	>100	$5.47\times 10^{-3}$
Intrinsic Pathway of Fibrin Clot Formation	>100	$2.97\times 10^{-6}$
Plasma lipoprotein assembly	>100	$3.56\times 10^{-2}$
Formation of Fibrin Clot (Clotting Cascade)	>100	$2.28\times 10^{-5}$
Post-translational protein phosphorylation	67.32	$1.62\times 10^{-8}$
Regulation of IGF transport and uptake by IGFBPs	58.09	$2.31\times 10^{-8}$
Platelet degranulation	48.61	$1.55\times 10^{-6}$
Response to elevated platelet cytosolic Ca^2+^	46.77	$1.46\times 10^{-6}$
Regulation of Complement cascade	28.06	$3.77\times 10^{-2}$
Complement cascade	25.73	$4.47\times 10^{-2}$
Platelet activation, signalling and aggregation	23.75	$4.8\times 10^{-5}$
Hemostasis	10.73	$6.55\times 10^{-4}$

**Table 5:** A summary of statistical samples.

Recruitment
74 patients
Number of follicular fluids tested (maximum 3 per patient)
213
Number of biological repeats
639 (213×3)

**Table 6:** Parameters used to ensure optimum sample preparation and instrument performance for SWATH-MS analysis.

Steps		
Protein fractionation for the library		high-pH RP-HPLC, Immunodepletion
Fractionation of peptides for quantitative analysis		No
Digestion method		FASP (trypsin)
Peptide purification		C18 Stage Tips
Method parameters LC		30 min, 8–40% buffer B
Parameters	Data-dependent acquisition (DDA) MS	400–1000 Da, 250 ms
	MS/MS	100–1500 Da, 100 ms
	Cycle time	2.3 s
SWATH-MS Method parameters	MS	400–1000 Da, 50 ms
	MS/MS	10–1500 Da, 40 ms
	Cycle time	1.1 s
Transmission windows		25 window variables in range 400–1000 Da
Results		
Total number of proteins identified in the experiments		2182
Number of proteins identified in fractions HMWF/LMWF		2177/14
Number of proteins identified after ultrafiltration		129
Number of quantified proteins		484
Number of proteins quantified with CV < 20%		98

Abbreviations: FASP—Filter-Aided Sample Preparation; HMWF—High-Molecular-Weight Fraction; LC—Liquid Chromatography; LMWF—Low-Molecular-Weight Fraction; MS—Mass Spectrometry; MED-FASP—Multi-Enzyme; RP-HPLC—Reversed-Phase High-Performance Liquid Chromatography; SWATH-MS—Sequential Window Acquisition of all Theoretical Mass Spectra.

## Data Availability

The datasets presented in this article are not readily available because the data are part of an ongoing study. Requests to access the datasets should be directed to janusz.przewocki@ug.edu.pl.

## Abbreviations

The following abbreviations are used in this manuscript:

AFC	antral follicles count
AMH	anti-Mullerian hormone
BMI	body mass index
DDA	Data-dependent acquisition
DIA	Data-independent acquisition
DHEAS	dehydroepiandrosterone-sulfate
E2	oestradiol
FASP	Filter-Aided Sample Preparation
FF	follicular fluid
FSH	follicle-stimulating hormone
FC	fold change
hCG	human chorionic gonadotropin
HDL	high-density lipoprotein
HMWF	High-Molecular-Weight Fraction
IVF	In vitro fertilisation
LC	Liquid Chromatography
LDL	low-density lipoprotein
LH	luteinising hormone
LMWF	Low-Molecular-Weight Fraction
MS	Mass Spectrometry
MED-FASP	Multi-Enzyme Digestion Filter-Aided Sample Preparation
RP-HPLC	Reversed-Phase High-Performance Liquid Chromatography
RFECV	Recursive Feature Elimination with Cross-Validation
SHBG	sex hormone-binding globulin
SWATH-MS	Sequential Window Acquisition of all Theoretical Mass Spectra
VLDL	very low-density lipoprotein
